# Exploring the effects of post operative hyperoxic intermittent stimuli on reticulocyte levels in cancer patients: a randomized controlled study

**DOI:** 10.1186/s44158-024-00179-x

**Published:** 2024-07-08

**Authors:** Maher Khalife, Michele Salvagno, Maurice Sosnowski, Costantino Balestra

**Affiliations:** 1 Institut Jules Bordet, Anaesthesiology, H.U.B, Brussels, Belgium; 2 Department of Intensive Care, Hôpital Universitaire de Bruxelles (H.U.B), 1070 Brussels, Belgium; 3 Environmental, Occupational & Ageing “Integrative Physiology” Laboratory, Haute Ecole Bruxelles-Brabant, Brussels, Belgium

**Keywords:** Normobaric hyperoxia, Normobaric oxygen paradox, Transfusion alternatives, Relative hypoxia

## Abstract

**Background:**

Anemia is common among hospitalized critically ill and surgical oncological patients. The rising incidence of cancer and aggressive treatments has increased the demand for blood products, further strained by a dwindling donor pool. The normobaric oxygen paradox (NOP) has emerged as a potential avenue to increase EPO levels. While some studies support its efficacy, research remains limited in clinical settings. This study aims to assess the effectiveness of a NOP protocol in stimulating erythropoiesis, as measured by changes in reticulocyte counts, in cancer patients undergoing abdominal surgeries.

**Methods:**

This is a post hoc analysis of a prospective, single-center, controlled, randomized study. A total of 49 patients undergoing abdominal surgery were analyzed at the Institut Jules Bordet. Adult patients admitted to the intensive care unit (ICU) for at least 24 h were enrolled, excluding those with severe renal insufficiency or who received transfusions during the study period. Participants were randomized into two groups: a normobaric oxygen paradox (OXY) group who received 60% oxygen for 2 h on days 1, 3, and 5 post-surgery and a control (CTR) group who received standard care. Data on baseline characteristics, surgical details, and laboratory parameters were collected. Statistical analysis included descriptive statistics, chi-square tests, *t*-tests, Mann–Whitney tests, and linear and logistic regression.

**Results:**

The final analysis included 33 patients (median age 62 [IQR 58–66], 28 (84.8%) males, with no withdrawals or deaths during the study period. No significant differences were observed in baseline surgical characteristics or perioperative outcomes between the two groups. In the OXY group (*n* = 16), there was a significant rise (*p* = 0.0237) in the percentage of reticulocyte levels in comparison to the CTR group (*n* = 17), with median values of 36.1% (IQR 20.3–57.8) versus − 5.3% (IQR − 19.2–57.8), respectively. The increases in hemoglobin and hematocrit levels did not significantly differ between the groups when compared to their baselines’ values.

**Conclusions:**

This study provides preliminary evidence supporting the potential of normobaric oxygen therapy in stimulating erythropoiesis in cancer patients undergoing abdominal surgeries. While the OXY group resulted in increased reticulocyte counts, further research with larger sample sizes and multi-center trials is warranted to confirm these findings.

**Trial registration:**

The study was retrospectively registered under NCT number 06321874 on The 10th of April 2024.

## Background

Anemia is a common occurrence in hospitalized patients and often leads to the necessity of red blood cell transfusions (RBCT), which come with an increased risk of side effects [1, 2]. Therefore, finding ways to reduce the incidence of anemia and decrease the need for transfusions is of great interest in clinical practice. Patients who have undergone surgery frequently suffer from anemia [3]. This prevalence is higher in oncological patients [4], where both the disease and the chronic kidney impairment that the patients can develop, as well as chemotherapy and other treatments, can contribute to a decrease in red blood cell (RBC) levels. Furthermore, cancer patients usually require multiple units of blood if they need transfusions [4, 5]. The increasing demand for RBCT in recent years, particularly due to advances in aggressive cancer treatments and the rising incidence of cancer, presents a significant challenge. This challenge is compounded by the ongoing risks associated [6, 7].

Erythropoietin (EPO) is a crucial hormone that initiates the generation of RBC by stimulating progenitor cells in the red bone marrow [8, 9]. For anemia treatment in cancer patients, human recombinant erythropoietin is often used [10, 11]. However, its application is constrained due to high costs, possible side effects, and limited availability in some regions. This has encouraged research and clinical exploration for alternative methods.

Oxygen is widely used for various purposes across different fields. In this context, one of its areas of application is its influence on EPO [12]. Indeed, EPO regulation is largely affected by hypoxia mainly in kidney tissues. Consequently, the “normobaric oxygen paradox” (NOP) might enhance its production [13]. NOP involves intermittent exposure to higher oxygen levels at normal atmospheric pressure. This has been shown to enhance the production of erythropoietin and raise hemoglobin levels by increasing reticulocyte counts [14]. Its potential for therapeutic use extends across various medical fields. This effect appears to be driven by intricate cellular processes balancing reactive oxygen species (ROS) production and natural antioxidants. Periodic hyperoxia reduces the ROS/scavenger ratio to levels mimicking hypoxic conditions, leading to the release of HIF-1α, initiating the EPO gene expression cascade, and thus enhancing EPO production among other stimuli [15]. While some research supports the effectiveness of pure oxygen inhalation in NOP-induced anemia treatment, results vary, especially in healthy individuals, indicating that NOP’s impact might be dependent on dosage and duration [16–19]. Yet, the ideal timing and strength of the oxygen stimulus remain undefined. There is a scarcity of clinical studies in this field, and the precise mechanism of NOP is still not completely understood.

Our primary research goal is to ascertain whether a NOP protocol can increase reticulocyte counts, indicating a rise in EPO production, among cancer patients undergoing abdominal surgeries.

## Methods

This investigation was retrospectively conducted on data from a prospective, single-center, single-blind, controlled, randomized trial, performed at the Institut Jules Bordet (Belgium), from February to November 2011, and registered with Clinical Trials (NCT 06321874). It conformed to the Helsinki Declaration and was approved by the local ethics committee at the Institut Jules Bordet, Belgium (approval number CE1805). Written informed consent was secured from all participants.

### Inclusion and exclusion criteria

The study included adult patients (aged 18 or older), eligible for abdominal oncological surgery. Exclusion criteria were severe renal insufficiency (glomerular filtration rate (GFR) < 60 ml/min and creatinine serum level > 2 mg/dl), transfusion of red blood cells, plasma or plaquettes during the perioperative period, bleeding requiring repeated transfusions during or after surgery, severe respiratory syndrome necessitating continuous oxygen, and intolerance to an oxygen mask.

### Randomization process

Randomization was conducted post-surgery in a 1:1 ratio across groups: normobaric oxygen paradox (OXY) group, and a control (CTR) group. The OXY group received 60% oxygen for 2 h on days 1, 3, and 5 post-surgery using a venturi mask (with a 60% oxygen dedicated adapter and at 10 l/min adjusted by the mean of a flow meter). The CTR group did not receive oxygen therapy during the post-operatory period starting from day 1. All patients could receive oxygen during the surgery and the first 24 h post-surgery (day 0) as needed. The floor nursing along with the supervisor’s protocol ensured that the patients received the appropriate oxygen flow and the correct adapter. The belonging to one group or the other was blinded to the laboratory staff.

### Sample size calculation

The power calculation and sample size determination were performed in advance using G*Power 3.1 software. This was based on previously published data [18] and established parameters with significance level (α) at 0.05, effect size = 0.70, alpha error = 0.05, and power = 0.90. Given that the protocol involved a relative increase compared to the baseline (with each subject serving as their own control), 24 participants were deemed necessary for this study to evaluate the magnitude of the treatment effect.

### Medical treatments

In pre-surgery, all patients were administered an oral benzodiazepine. General anesthesia was employed, potentially combined with epidural techniques and standard anesthesia monitors were used. The choice of anesthesia technique was at the discretion of the anesthetist. During surgery, patients received 40–50% FiO2 ventilation and were extubated at the surgery’s end. Post-surgery, patients were admitted to the ICU for at least 24 h, receiving oxygen if saturation dropped below 98%. The oxygen protocol commenced 24 h after ICU admission (day 1). The transfusion threshold was set at 8 g/dl along with clinical signs.

### Study outcomes

The primary endpoint was the percentage change in reticulocyte count from baseline to day 6 within each group. Secondary endpoints included hematocrit and hemoglobin levels measured at baseline and on day 6.

### Parameters collected and statistical analysis

A comprehensive set of clinical data, including information on age, gender, tobacco use, ASA score (American Society of Anesthesiologists score), and body mass index (BMI) have been collected. Additionally, surgery-specific data was gathered, encompassing details about the type of surgery performed and its duration. Laboratory data obtained on both baseline prior to surgery (D0) and day 6 postoperatively (D6) included measurements of reticulocyte count, hemoglobin levels, hematocrit levels, urea, creatinine, ferritin, transferrin saturation, and iron levels. Blood samples were collected at 8 AM in all patients. Any postoperative complications that arose were carefully documented. Blood loss during surgery was calculated by weighing compresses and measuring bowel aspiration. Post-surgery blood loss was determined from the drainage output.

Descriptive statistics were computed for all study variables. Categorical data are presented as both as numbers and as percentages. Continuous data are presented as mean (± standard deviation) or median (25th–75th percentiles), according to the distribution pattern of each variable. Differences between groups were assessed using a χ-square test for categorical variables. For normally distributed continuous variables, we used the *t*-Student test, and for asymmetrically distributed continuous variables, we applied the Mann–Whitney test. To assess changes over time, mean percentage changes in reticulocyte counts, hemoglobin levels, and hematocrit levels from their respective baseline values were calculated. We used a binary logistic regression to study the association between the two groups related to oxygen administration vs. control and the increase of the reticulocyte count. *p*-values < 0.05 were considered significant. Linear regression was performed using the least squares method. Statistical analyses were performed using Jamovi (v 2.3) and Prism (v 10.2).

## Results

A total of 49 patients undergoing abdominal surgery were evaluated for the aim of this study; 16 subjects were excluded following the exclusion criteria. A total of 33 patients were included in the final analysis. No patients withdrew for medical reasons, and no patients died during the study period. The baseline characteristics of the two groups are detailed in Table 1.

**Table 1:** Control group (CTR) compared to the hyperoxic stimulus group (OXY) in baseline and perioperative characteristics. Values are expressed as median and interquartile range (IQR) or number of patients and percentages on the total of the group

Characteristics	CTR *n* = 17 (52%)	OXY *n* = 16 (48%)	*p*
Age (years)	62 (59–66)	61 (58–71)	0.87
Male sex	13 (76%)	15 (94%)	0.17
Weight (kg)	83 (79–95)	86 (74–92)	0.93
Body surface (m^2^)	1.98 (1.92–2.08)	1.98 (1.85–2.07)	0.99
BMI (kg/m^2^)	28.0 (25.1–30.9)	29.0 (23.5–31.0)	0.90
ASA 3	15 (88%)	14 (88%)	0.99
ASA 2	2 (12%)	1 (6.2%)	
ASA 1	0	1 (6.2%)	
Comorbidities			
Smoke	2 (12%)	5 (31%)	0.22
Hypertension	6 (35%)	8 (50%)	0.39
Diabetes	4 (24%)	0	0.10
Anticoagulants	0 (100%)	1 (6.2%)	0.48
Stroke	1 (5.9%)	1 (6.2%)	0.99
Hyperlipidemia	10 (59%)	10 (62%)	0.83
Aspirin	0	4 (25%)	0.04*
Surgery			
Surgery length (minutes)	324 (288–385)	333 (264–390)	0.94
Crystalloids (mL)	2500 (2000–3000)	2050 (2000–3625)	0.96
Diureses (mL)	275 (100–500)	550 (125–688)	0.66
Blood loss during surgery (mL)	305 (138–436)	350 (200–750)	0.62
Blood loss at the 6th day (mL)^a^	640 (200–1040)	1043 (563–1508)	0.17
Laboratory data			
Reticulocyte count day 0	67.7 (57.8–81.0)	61.7 (56.4–73.8)	0.50
Reticulocyte count day 6	68.0 (61.1–77.2)	89.7 (61.6–119.5)	0.12
Hb (g/dl) day 0	14.5 (13.6–15.6)	15.3 (13.2–15.7)	0.73
Hb (g/dl) day 6	13.9 (11.7–14.4)	12.1 (9.7–13.1)	0.04*
Hb difference day 6–day 0 in %	− 11.2 (− 17.4 to − 5.03)	− 18.8 (− 23.1 to − 10.8)	0.234
Hct % day 0	43.2 (40.4–45.7)	44.8 (39.4–45.5)	0.95
Hct % day 6	39.9 (33.4–42.4)	35.5 (29.6–41.5)	0.10
Urea mg/dl day 0	32.0 (30.2–39.8)	43.0 (40.0–46.8)	0.01*
Urea mg/dl day 6	27.0 (21.0–31.8)	35.0 (26.0–38.7)	0.07
Creatinine mg/dl day 0	0.9 (0.9–1.0)	1.0 (0.9–1.1)	0.56
Creatinine mg/dl day 6	30 0.7 0.8 (0.7–1.0)	0.9 (0.8–1.1)	0.12
Ferritin g/dl day 0	160.0 (66.0–331.0)	274.0 (146.2–287.7)	0.53
Ferritin g/dl day 6	297.0 (241.8–504.3)	464.0 (266.8–551.2)	0.45
Transferrin saturation % day 0	36.0 (20.5–46.0)	32.0 (26.7–45.7)	0.76
Transferrin saturation % day 6	12.0 (8.3–21.7)	12.0 (9.3–15.8)	0.64
Outcomes			
LOS hospital (days)	9 (7–14)	7 (6–10)	0.08
LOS ICU (days)	1 (1–2)	1 (1–3)	0.99
Infections	4 (24%)	1 (6.2%)	0.34
Complications	4 (24%)	0	0.10

*BMI* body mass index, *ASA* American Society Anesthesia, *Hb* hemoglobin, *Hct* hematocrit, *LOS* length of stay, *ICU* intensive care unit

^*^Significant result

^a^Post-surgery blood loss from drainage output

Between the CTR group (*n* = 17) and the OXY group (*n* = 16), the CTR group reported more infections (4 vs. 1, *p* = 0.34), more complications (4 vs. 0, *p* = 0.10), and a longer hospital stay (9 vs. 7 days, *p* = 0.08), although these differences did not reach statistical significance, as shown in Table 1. The only characteristic showing a statistically significant difference was the use of aspirin, with 4 cases in the OXY group versus none in the CTR group (*p* = 0.04).

**Fig. 1:**
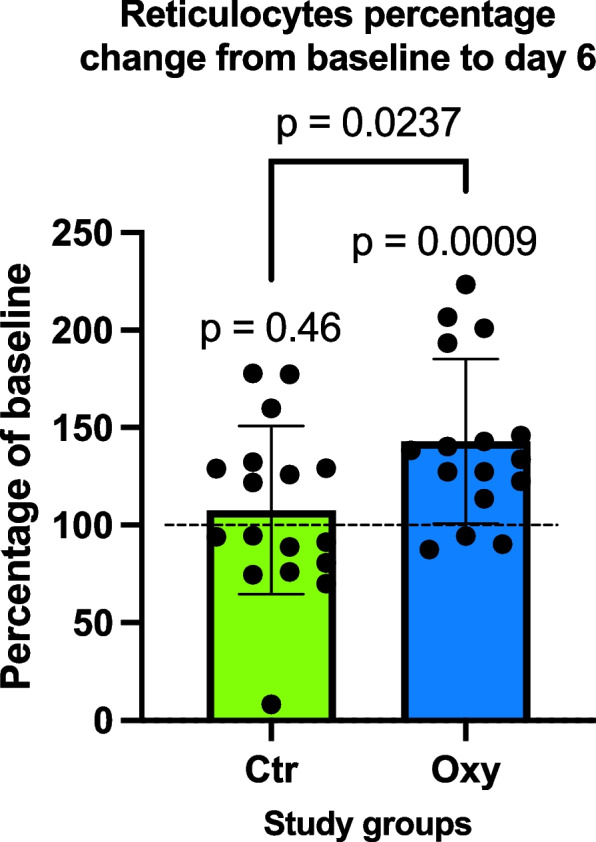
Boxplot comparison of reticulocyte percentage change from baseline between control (CTR) and oxygen therapy (OXY) study groups

The analysis revealed a significant difference in the percentage change of reticulocytes from D6 to baseline between the two groups, with a Mann–Whitney *U* test yielding a *p*-value equal to 0.0237, as described in Fig. 1, with median values of 36.1% (IQR 20.3–57.8) versus − 5.3% (IQR − 19.2–57.8), respectively. When dichotomizing this parameter, it was found that 13 patients in the OXY group (81.3%) experienced an increase in their reticulocyte count from baseline (percentage change greater than 100%). In contrast, only 3 patients in the CTR group (17.6%) exhibited an increase from their baseline reticulocyte values, and this difference was statistically significant (*p* = 0.04).

The reticulocyte count is presented as a percentage change from baseline levels, measured on day 6 (D6) post-intervention. This difference between the two groups is statistically significant, with a *p*-value = 0.0237. Outliers are represented by dots outside of the whiskers.

**Fig. 2:**
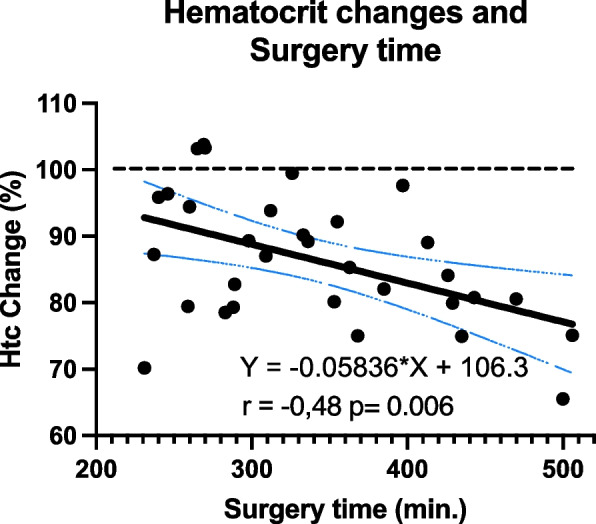
Scatter plot with trend line illustrating the relationship between surgery duration and hematocrit percentage change from baseline. The plot shows individual patient data points with a fitted regression line indicating a significant decrease of hematocrit levels with increasing duration of surgery. The shaded area represents the 95% confidence interval for the regression line

Through a univariate logistic regression analysis, it was determined that being in the OXY study group was associated with an increased likelihood of experiencing reticulocyte count growth (OR 4.88 [1.01–23.57]). No other factors considered in univariate analysis were found to be significant risk factors. Finally, the duration of surgery exhibited a statistically significant decrease to be negatively correlated with the difference in hematocrit levels between D6 and baseline (correlation coefficient: *r* =  − 0.48, *p* = 0.006), as illustrated in Fig. 2.

## Discussion

The findings from this study shed light on the potential effectiveness of normobaric oxygen use in mitigating anemia in patients undergoing abdominal cancer surgery. Anemia is commonly observed both before and after these surgical procedures, often leading to allogeneic transfusions due to significant blood loss or critical vital signs. Previous studies have associated transfusions with increased mortality, prolonged hospital stays, and higher morbidity rates [20, 21]. In our research, we excluded from the statistical analysis those patients who received transfusions either during or after surgery, as transfusions can influence reticulocyte production [22, 23].

A significant rise in reticulocyte count was noted in the OXY group post-surgery, supposing an increase in EPO production, consistent with the NOP hypothesis [18]. Following this hypothesis, an intermittent hyperoxic stimulus (characterized by brief periods of elevated oxygen concentration, alternating with lower levels, and repeated over several days or multiple times within a single day) has been found to have a paradoxical effect, i.e., cells react as if experiencing low oxygen conditions, with increased production of erythropoietin (EPO) [13].

However, the role of NOP in enhancing reticulocyte and red blood cell production is still debated [14, 24–27]. It is theorized that a minimum of 40% O2 may be necessary to initiate NOP, with 100% oxygen showing varied results. In our study, we used a 60% inspired O2 concentration to assess its impact. Both the groups showed higher absolute reticulocyte counts, potentially due to a general increase in reticulocyte count and the administration of preoperative oxygen (typically FiO2 30–50%), followed by decreased oxygen levels provided after surgery, thus creating a hyperoxic-hypoxic stimulus in all participants. However, the OXY group alone showed a statistically significant mean percentage change in reticulocyte counts from baseline to day 6, indicating that a larger oxygen gradient in this group compared to the CTR group may have contributed to this observation. Very recent data show a possible beneficial and faster effect when using even lower levels of oxygen (smaller gradient), which favors HIFs translocation without triggering important oxidative stress [26, 28].

Our results align with the proposed mechanism of NOP in enhancing endogenous EPO production. The notable increase in reticulocyte counts in the OXY group, relative to the control group, highlights NOP’s potential in stimulating erythropoiesis. This supports the theory that intermittent hyperoxia may lead to a rise in EPO production upon returning to normoxia, a process thought to involve hypoxia-inducible factors and ROS neutralization by antioxidants like glutathione [13]. The control group did not show a significant increase in reticulocyte counts 1-week post-surgery compared to the OXY group, which aligns with the existing literature [18]. The inflammatory response to surgery, potentially increasing hepcidin synthesis and affecting iron availability for EPO action, could have blunted the reticulocyte increase in both groups. Despite this impairment, a positive reaction was present only in the OXY group overcoming the inflammatory possible interference. Moreover, only three patients in the CTR group (17.6%) exhibited an increase from their baseline reticulocyte values, and this difference was statistically significant (*p* = 0.04).

Nevertheless, new data are encouraging and show repetitive oxygen exposures elicit pro and anti-inflammatory effects [29, 30].

Recent findings show that short-term exposure to high oxygen levels can induce a complex biological response involving oxidative stress and inflammation [28, 31–35], and it seems that the NOP mechanism, to be profitable, depends on a subtle, dynamic equilibrium involving antioxidants such as NRF2, oxidative stress (ROS), and inflammatory markers such as NFKB [26, 28]. When the equilibrium is more set toward oxidative and pro-inflammatory responses, the NOP response is blunted, and the positive rebound reactions need more recovery time. On the contrary, when the equilibrium tends more toward the “pure” antioxidant reactions elicited, the rebound response is higher and more rapid. This intricate, complex phenomenon warrants more research to define the right dose (oxygen exposure, FiO_2_, and duration) and the correct adapted intermittency (recovery time needed to elicit the optimal response) between the doses [26, 36]. Indeed, a single exposure [33] and intermittent (i.e., repetitive) hyperoxic stimuli [36, 37] have been shown to be associated with a different response. These insights could inform medical practices involving oxygen therapy, such as preoperative oxygenation or treatments for oxidative stress conditioning [14, 29, 37].

Our results indicate a significant difference in hemoglobin values between the groups (*p* = 0.04), with the CTR group showing higher levels. This difference may be due to the greater blood loss observed in the OXY group. At this stage, we cannot rule out that blood loss may have contributed to the increase in reticulocytes observed in the OXY group. In contrast, the reticulocyte increasing in the CTR group, if attributed to blood loss, was not as pronounced. Therefore, we hypothesize that the effect of oxygen outweighed the impact of blood loss on the reticulocyte response. Moreover, the percentage change in hemoglobin levels from day 0 to day 6 did not show a statistically significant difference between the two groups (*p* = 0.234).

Additionally, we observed a negative statistically significant correlation between the duration of surgery and hematocrit changes, supporting the hypothesis that longer surgical times might be associated with greater intraoperative blood loss or hemodilution, which could subsequently affect postoperative recovery and mitigate NOP benefits. In other words, it might affect erythropoiesis or erythrocyte turnover. This finding, coupled with the impact of surgery duration on reticulocyte dynamics, highlights the need for careful surgical planning and possibly using intermittent hyperoxia as a modifiable factor to mitigate adverse outcomes in longer surgeries. Possible NOP-eliciting methods may even be developed during long surgery procedures. It is obvious that longer surgeries also lead to more oxygen exposure, which may temporarily suppress EPO synthesis and reticulocyte production and could contribute to these findings.

Furthermore, longer surgical times could also increase systemic acute inflammatory response [38], thus promoting the anemia of inflammation [39]. The impact of surgical duration aligns with the known anemic effect of surgeries caused by surgical-related inflammation other than fluid administration [40].

The findings of these studies could have important clinical implications, particularly considering the high prevalence of anemia in oncological patients and the risks and limitations associated with blood transfusions. NOP offers a promising, non-invasive, and cost-effective alternative, especially in settings with limited access to blood products or recombinant EPO [13].

Moreover, the assessments of the cost-effectiveness of EPO administration show that, in some perioperative settings, EPO provides minimal overall improvement in patient health, and the limited benefits it offers come at a very high cost [41].

However, this study have several limitations. The small sample size, although sufficient, and the single-center design may also restrict the generalizability of our findings. Additionally, the absence of significant differences in hemoglobin with respect to their baseline value and hematocrit levels suggests that while NOP may encourage erythropoiesis, its effect on overall anemia management is more nuanced and requires further exploration. This mirrors the results in healthy volunteers, where increased EPO levels did not coincide with a rise in hemoglobin. This may be understandable because it should take more time to reach an increase in hemoglobin, as well as the change in the dose and the time between the doses provided, as was shown by Cimino et al. [42] after administration of 30 min of pure oxygen every other day for 10 days, or by De Bels et al. [14] every other day opposed to every day. The lack of baseline anemia, short treatment duration, and brief period for measuring hemoglobin in our study likely contributed to the absence of notable hemoglobin changes. Another limitation of the study is that it includes different types of surgical procedures. Hence, it is important to acknowledge that this factor may lead to varying physiological responses post-surgery.

Finally, although blood loss was higher in the OXY group, there was no significant difference compared to the CTR group. This could be attributed to the higher aspirin use in the OXY group, the extent of tissue trauma, and potentially to the surgery-related third fluid space involvement which we could not control [40].

The preliminary evidence provided by this study supports the potential benefits of hyperoxic therapeutic stimuli in enhancing erythropoietic response and possibly improving surgical outcomes. It could thus be incorporated into the Enhanced Recovery After Surgery (ERAS) principles and protocols [43] by contributing to enhancing patient outcomes and lowering healthcare costs while promoting a quicker recovery. Future prospective studies, ideally randomized controlled trials with larger cohorts, are needed to further elucidate the role of hyperoxia in surgical settings and to confirm these findings. This could eventually lead to a shift in perioperative management strategies to optimize patient outcomes through targeted oxygen therapy.

## Conclusions

In conclusion, our study presents preliminary evidence supporting the potential role of relative hypoxia induced by an oxygen gradient in enhancing erythropoiesis by stimulating reticulocyte production, i.e., the normobaric oxygen paradox, in post-operative cancer patients. These findings open avenues for further research and could have significant implications in managing anemia in surgical patients, offering a safer and more accessible alternative to mitigate blood transfusions and recombinant EPO therapy. Nevertheless, these results warrant additional randomized trials involving a larger population to investigate their influence on hemoglobin production and the extent of effects on the oxygen gradient.

## Data Availability

The datasets used and/or analyzed during the current study are available from the corresponding author on reasonable request.

## Abbreviations

NOP: Normobaric oxygen paradox
EPO: Erythropoietin
ICU: Intensive care unit
RBCT: Red blood cell transfusions
RBC: Red blood cell
ASA: American Society of Anesthesiologists score
BMI: Body mass index
Hb: Hemoglobin
Hct: Hematocrit
